# Severe Acute Respiratory Syndrome Coronavirus 2 (SARS-CoV-2) Infectivity by Viral Load, S Gene Variants and Demographic Factors, and the Utility of Lateral Flow Devices to Prevent Transmission

**DOI:** 10.1093/cid/ciab421

**Published:** 2021-05-11

**Authors:** Lennard Y W Lee, Stefan Rozmanowski, Matthew Pang, Andre Charlett, Charlotte Anderson, Gareth J Hughes, Matthew Barnard, Leon Peto, Richard Vipond, Alex Sienkiewicz, Susan Hopkins, John Bell, Derrick W Crook, Nick Gent, A Sarah Walker, Tim E A Peto, David W Eyre

**Affiliations:** 1 Nuffield Department of Medicine, University of Oxford, United Kingdom; 2 Department of Health and Social Care, UK Government, London, United Kingdom; 3 Public Health England, London,United Kingdom; 4 Public Health England, Porton Down,United Kingdom; 5 NIHR Oxford Biomedical Research Centre, University of Oxford, Oxford,United Kingdom; 6 NIHR Health Protection Research Unit in in Healthcare Associated Infections and Antimicrobial Resistance, University of Oxford, Oxford, United Kingdom; 7 Big Data Institute, Nuffield Department of Population Health, University of Oxford, Oxford, United Kingdom

**Keywords:** infectivity, contact tracing, SARS-CoV-2, lateral flow device, B.1.1.7 variant

## Abstract

**Background:**

How severe acute respiratory syndrome coronavirus 2 (SARS-CoV-2) infectivity varies with viral load is incompletely understood. Whether rapid point-of-care antigen lateral flow devices (LFDs) detect most potential transmission sources despite imperfect clinical sensitivity is unknown.

**Methods:**

We combined SARS-CoV-2 testing and contact tracing data from England between 1 September 2020 and 28 February 2021. We used multivariable logistic regression to investigate relationships between polymerase chain reaction (PCR)-confirmed infection in contacts of community-diagnosed cases and index case viral load, S gene target failure (proxy for B.1.1.7 infection), demographics, SARS-CoV-2 incidence, social deprivation, and contact event type. We used LFD performance to simulate the proportion of cases with a PCR-positive contact expected to be detected using 1 of 4 LFDs.

**Results:**

In total, 231 498/2 474 066 (9%) contacts of 1 064 004 index cases tested PCR-positive. PCR-positive results in contacts independently increased with higher case viral loads (lower cycle threshold [Ct] values), for example, 11.7% (95% confidence interval [CI] 11.5–12.0%) at Ct = 15 and 4.5% (95% CI 4.4–4.6%) at Ct = 30. B.1.1.7 infection increased PCR-positive results by ~50%, (eg, 1.55-fold, 95% CI 1.49–1.61, at Ct = 20). PCR-positive results were most common in household contacts (at Ct = 20.1, 8.7% [95% CI 8.6–8.9%]), followed by household visitors (7.1% [95% CI 6.8–7.3%]), contacts at events/activities (5.2% [95% CI 4.9–5.4%]), work/education (4.6% [95% CI 4.4–4.8%]), and least common after outdoor contact (2.9% [95% CI 2.3–3.8%]). Contacts of children were the least likely to test positive, particularly following contact outdoors or at work/education. The most and least sensitive LFDs would detect 89.5% (95% CI 89.4–89.6%) and 83.0% (95% CI 82.8–83.1%) of cases with PCR-positive contacts, respectively.

**Conclusions:**

SARS-CoV-2 infectivity varies by case viral load, contact event type, and age. Those with high viral loads are the most infectious. B.1.1.7 increased transmission by ~50%. The best performing LFDs detect most infectious cases.

## Teaser

In 2,474,066 contacts of 1,064,004 SARS-CoV-2 cases, PCR-positive tests in contacts increased with higher index case viral loads, the B.1.1.7 variant and household contact. Children were less infectious. Lateral flow devices detect 83.0-89.5% of infections leading to onward transmission

The global impact of severe acute respiratory syndrome coronavirus 2 (SARS-CoV-2) is profound [1]. There is widespread ongoing transmission despite control efforts predominantly focused on quarantining symptomatic cases and population-level self-isolation [2]. The emergence of potentially more transmissible variants, such as B.1.1.7 [3] in the United Kingdom, has hampered control. However, vaccination offers the prospect of reduced disease and transmission [4].

Intermittent social distancing and self-isolation measures have been imposed in many countries [5, 6]. Additional self-isolation measures for “contacts” exposed to SARS-CoV-2 vary but generally last 7–14 days [7]. Although reducing transmission, quarantine/isolation measures have indirect effects on economic productivity, well-being [8] and non-coronavirus disease 2019 (COVID-19)-related excess deaths [9–11]. Not all SARS-CoV-2 exposure leads to infection; in some settings only 5–7% of contacts develop COVID-19 [12, 13] and modeling suggests ~15% of individuals are responsible for most SARS-CoV-2 transmission [14]. Using isolation selectively for the most infectious could lessen its collateral impacts [12, 13].

Several assays for infectivity have been proposed including functional (animal and cell culture) and nucleic acid (viral sub-genomic messenger RNA [mRNA]) models [15]. Detection of viral protein (antigen) by lateral flow devices (LFDs) has been more closely linked to viral culture infectivity than polymerase chain reaction (PCR) measurements [16]. However, few of these surrogate measures have been convincingly demonstrated to predict the real-world likelihood of transmission.

Here we use data from England’s national contact tracing and testing programs to explore the relationship between infectivity and SARS-CoV-2 viral load, as measured by PCR cycle threshold (Ct) values. We identify demographic factors associated with infectivity and the impact of the B.1.1.7 variant. We apply our results to a population of PCR-positive individuals to estimate the proportion of infectious individuals detected by viral antigen LFDs under a range of performance conditions.

## METHODS

Data from community and hospital PCR testing in England between 1 September 2020 and 28 February 2021 were linked with national contact tracing data by the UK Government Department of Health and Social Care. De-identified data extracts included for PCR-confirmed cases and their contacts: the nature of the contact events, demographic details (age, sex, ethnicity), if symptoms were present for cases and the timing of testing relative to symptom onset, and test results.

### Cases and Contacts

We defined index cases as SARS-CoV-2 PCR-positive individuals with a community-based test performed by 3 high-throughput national testing facilities (Milton Keynes, Alderley Park, Glasgow), which reported Ct values indicating viral load. Contacts of index cases were defined as all individuals notified to the national contact tracing service from the day of the index cases’ positive test until 10 days later with whom the index case had been in close proximity from 48 hours before their symptom onset to 10 days afterwards (further definitions in Supplementary Data).

### PCR Testing

Combined nose and throat swabs from index cases were processed using the same RNA extraction and Thermo Fisher TaqPath PCR platform in each laboratory (targeting S and N genes, and ORF1ab; see Supplementary Data, including estimation of viral load from Ct values). Only the first positive result per person was included. Index cases without available Ct values were excluded. The B.1.1.7 variant contains a Δ69–70 deletion, resulting in S gene target failure (SGTF). From mid-December 2020 onwards, ≥99% of sequences with Δ69–70 were B.1.1.7 (see Supplementary Data) [17], allowing SGTF to be used as a proxy for B.1.1.7. Contacts could be tested PCR-positive through any community or hospital-based test as results were nationally reported.

### Statistical Analysis

We determined factors associated with PCR-positive results in contacts, including demographics, viral load, and SGTF status of the index case. To identify outcomes most likely representing onward transmission from the index case rather than a third party, we excluded contacts named by more than one index case. We also restricted to positive test results obtained 1–10 days following the index case’s test date, that is, the period when the index case may have been infectious, excluding earlier results in contacts to avoid contacts who were the source for the index case’s infection. Given these exclusions, the absolute proportion of contacts testing PCR-positive cannot be interpreted as a secondary attack rate. In contacts with >1 PCR test within the follow-up window, all were considered to identify positive results.

We used multivariable logistic regression to investigate associations between PCR-confirmed infection in contacts (including contacts whether or not they had PCR tests) and the index case’s Ct value and SGTF status, the contact event nature, the case’s demographics, and incidence and social deprivation index at the contact’s home location. We did not adjust for case symptoms, as these may be mediators of viral load’s effect on transmission. We used splines to account for non-linearity in continuous variables and screened for all pairwise interactions between main effects (see Supplementary Data).

We performed sensitivity analyses to test our restriction to contacts tested 1–10 days after each index case and including only contacts with PCR tests. We used unadjusted linear regression to investigate the proportion of the variation in Ct values in contacts explained by cases’ Ct values.

### Simulations of LFD Performance

We used our findings to estimate the proportion of potential transmission events where the source case would have been detected using an antigen LFD, using existing data on the performance of 4 LFDs: Innova, Deep Blue, Orient Gene, and Abbott [18]. For each source case we simulated a positive or negative LFD result by randomly drawing from the probability of an LFD being positive by the source case’s Ct value (Supplementary Figure 1). Each simulation was repeated 1000 times. Additionally, we ran simulations for a range of hypothetical LFD performances.

### Ethics

The study was conducted as part of national COVID-19 surveillance under the provisions of Section 251 of the NHS Act 2006 and therefore did not require individual patient consent. It was approved by Public Health England’s (PHE’s) Research Ethics and Governance Group (PHE’s research ethics committee), the UK COVID-19 LFD oversight group and NHS Test and Trace.

## RESULTS

**Figure 1.**
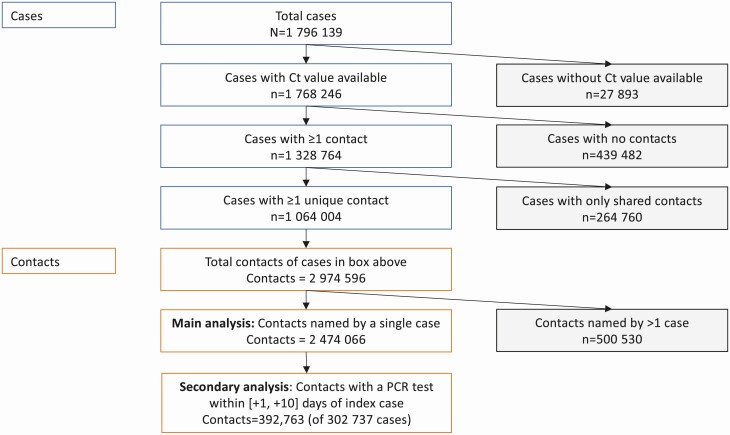
Index cases and contacts in England, 1 September 2020 to 28 February 2021. Abbreviations: Ct, cycle threshold; PCR, polymerase chain reaction.

In total, 3 577 246 SARS-CoV-2 PCR-positive results were available from England between 1 September 2020 and 28 February 2021. A total of 1 818 456 (51%) tests were performed by 3 national laboratories providing high-throughput community testing with a standardized PCR assay, yielding a first positive test per person in 1 796 139 individuals. Of these individuals, 27 893 (2%) were excluded as no Ct value was available, 439 482 (24%) had no recorded contacts, and 264 760 (15%) had only recorded contacts shared with other index cases, leaving 1 064 004 index cases in the analysis (Figure 1, Supplementary Table 1). In sum, 487 653 (46%) cases had SGTF, consistent with B.1.1.7, increasing to near 100% by 28 February 2021 (Supplementary Figure 2).

The 1 064 004 index cases had 2 974 596 contacts identified within 10 days of their test of whom 918 758 (31%) had a PCR test within ±10 days of the index case and 638 456 (21%) tested PCR-positive. The 2 474 066 (83%) contacts named only by a single case were included in the analysis; 231 498 (9%) tested PCR positive 1–10 days after the index case’s PCR-positive result, our main outcome measure, that is, consistent with possible transmission from the index case to the contact.

The median (IQR) age of cases and contacts was 36 (24–51) and 31 (16–49) years respectively, and 54% and 52% with available data were female (Table 1). Most contact events occurred within households (77.4%), followed by visits to households (8.3%), workplaces or education (8.0%), attending events or activities (5.3%), and outdoors (0.3%).

**Table 1.** Demographics and Characteristics of the Study Population

Variable	Case, n = 1 064 004^a^	Contact: Not PCR-positive Within 1–10 days, n = 2 242 569^a^	Contact: PCR-positive Within 1–10 days, n = 231 497^a^
Sex			
Female	560 557 (53%)	820 203 (37%)	114 837 (50%)
Male	476 967 (45%)	765 538 (34%)	99 539 (43%)
Not specified	26 480 (2.5%)	656 828 (29%)	17 121 (7.4%)
Age	36 (24–51)	30 (15–48)	37 (23–52)
Not available	9	720 544	17 853
Ethnic group			
Asian	128 218 (12%)	77 932 (3.5%)	9491 (4.1%)
Black	27 658 (2.6%)	17 167 (0.8%)	1874 (0.8%)
Mixed	27 263 (2.6%)	19 342 (0.9%)	2297 (1.0%)
Other	15 682 (1.5%)	9667 (0.4%)	1170 (0.5%)
White	728 265 (68%)	585 255 (26%)	78 363 (34%)
Not available	136 918 (13%)	1 533 206 (68%)	138 302 (60%)
Incidence at home address, per 100 000 population	355 (215–546)	348 (207–524)	375 (226–581)
Not available	4124	6444	493
Deprivation index at home address (lower = more deprived, of 32 844 areas)	14 465 (11 374–18 704)	14 465 (11 304–18 649)	14 593 (11 744–19 165)
Not available	4124	6444	493
Case symptomatic	969 942 (91%)		
Days from symptom onset to test in case where symptomatic	2 (1–3)		
Contact type			
Events/activities		137 805 (6.1%)	8919 (3.9%)
Household		1 718 674 (77%)	196 508 (85%)
Household visitor		189 637 (8.5%)	16 426 (7.1%)
Outdoors		8002 (0.4%)	317 (0.1%)
Work/education		188 451 (8.4%)	9327 (4.0%)
Days from case diagnosis to contact notification		2 (2–3)	2 (1–3)
Days from index case test to contact’s test where tested		2 (1–4)	3 (2–5)

Abbreviation: PCR, polymerase chain reaction.

^a^Frequency (%) or median (interquartile range [IQR]).

### Predictors of PCR-Positive Results in Contacts

On univariable analysis (Table 2, Supplementary Figures 3–6), PCR-positive tests in contacts were associated with lower case Ct values (ie, higher viral loads), SGTF in the index case, higher incidence in the local population, less social deprivation, white ethnicity and male sex. Household contacts were most likely to be PCR-positive. PCR-positive results were least frequent in contacts of children, with highest rates in contacts of older adults.

**Table 2.** Univariable and Multivariable Associations With the Proportion of Contacts Testing PCR Positive.

		Univariable			Multivariable		
Variable		OR	95% CI	*P* value	OR	95% CI	*P* value
Incidence contact’s home address, per 100 000 population^a^	50 (baseline)	1.00		<.001	Interaction with SGTF, see Supplementary Figure 7		
	100	1.10	1.09–1.11				
	200	1.25	1.24–1.26				
	400	1.25	1.24–1.26				
	600	1.42	1.41–1.43				
Deprivation score at contact’s home address (lower = more deprived)^a^	7000 (baseline)	1.00		<.001	1.00		<.001
	14 000	1.11	1.10–1.11		1.07	.92–1.24	
	21 000	1.26	1.25–1.27		1.16	1.00–1.35	
	28 000	1.25	1.20–1.25		1.14	.98–1.33	
Case Ct value (lower = higher viral load)^a^	10 (baseline)	1.00		<.001	Interaction with SGTF, see Figures 2 and 3		
	15	.81	.80–.81				
	20	.57	.57–.57				
	25	.44	.43–.44				
	30	.28	.28–.29				
SGTF	Wildtype (baseline)	1.00			Multiple interactions, see other rows		
	S gene variant	1.52	1.50–1.53	<.001			
Case sex	Female	1.00			Interaction with age, see Supplementary Figure 8		
	Male	1.04	1.03–1.04	<.001			
	Not specified	.73	.71–.75	<.001			
Case age^a^	30 years (baseline)	1.00		<.001	Interactions between SGTF and contact type, SGTF and age, contact type and age, see Figures 2 and 4		
	10 years	.71	.70–.72				
	50 years	1.34	1.33–1.34				
	70 years	1.40	1.38–1.41				
Contact event	Household (baseline)	1.00					
	Activities and events	.57	.55–.58	<.001			
	Household visitor	.76	.75–.77	<.001			
	Work or education	.43	.42–.44	<.001			
	Outside	.35	.31–.39	<.001			
Case ethnicity	White (baseline)	1.00			Interactions between ethnicity and SGTF, ethnicity and contact type, ethnicity and age, see Supplementary Figures 9 and 10		
	Asian	.74	.73–.75	<.001			
	Black	.67	.65–.69	<.001			
	Mixed	.81	.79–.83	<.001			
	Other	.78	.75–.81	<.001			
	Not available	.76	.75–.77	<.001			

Lower rates of PCR-positivity seen in cases without a documented sex possibly reflect incomplete contact tracing or poor data quality preventing appropriate linkage of these cases.

Abbreviations: CI, confidence interval; OR, odds ratio; PCR, polymerase chain reaction; SGTF, S gene target failure.

^a^Incidence, deprivation score, index case cycle threshold (Ct) value and case age are all fitted as nonlinear effects with 5 default-spaced knots, example values are shown, and univariable relationships plotted in Supplementary Figures 3–6. Multivariable results are presented with continuous variables set to their median value and categorical variables set to baseline, figures illustrating relationships with interactions are listed. See Supplementary Figure 11 for the multivariable relationship for deprivation score.

**Figure 2.**
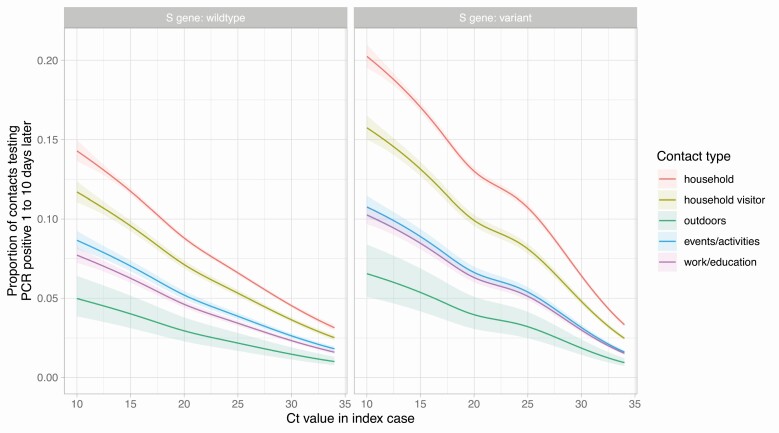
Relationship between PCR Ct value in cases and the proportion of their contacts with a PCR positive result, by contact type and S gene target failure. Model predictions are plotted after adjustment for index case age (set to the median value, 35 years), case ethnicity (set to White), index of multiple deprivation score at contact’s home address (set to median, 14 465), incidence at contact’s home address (set to median 350 cases per 100 000 population per week) and index case sex (set to female). Shaded area indicates the 95% confidence interval. Abbreviations: Ct, cycle threshold; PCR, polymerase chain reaction.

**Figure 3.**
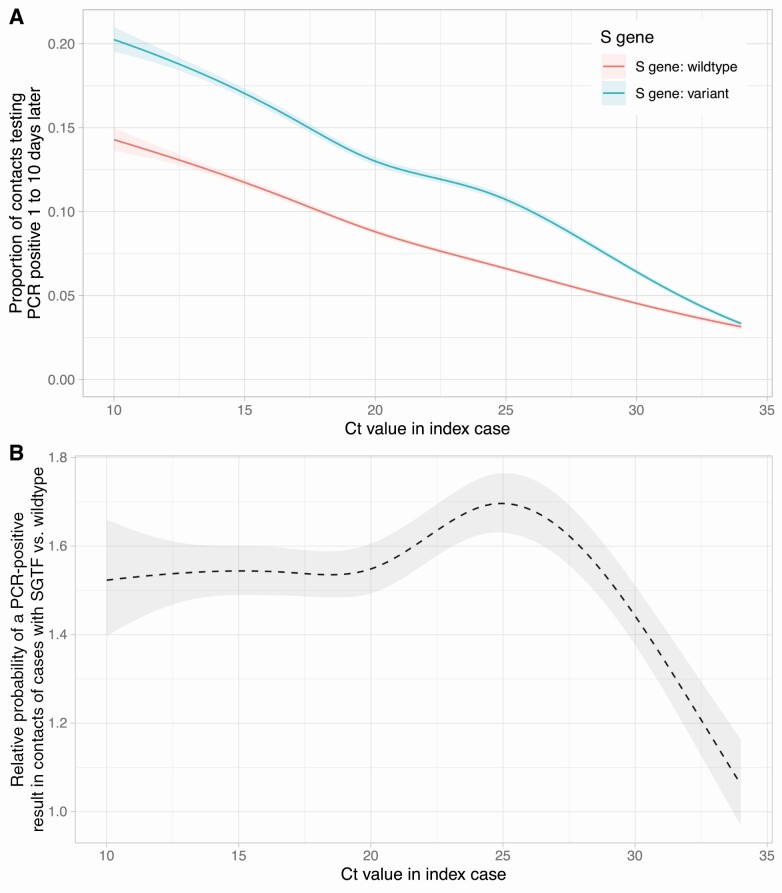
Relationship between PCR-positive results in contacts and index case Ct value and SGTF indicative of the B.1.1.7 variant. *A*, Proportion of contacts testing by PCR-positive. *B*, Ratio of the 2 lines from panel *A*, ie, the relative infectiousness of index cases with SGTF vs without SGTF. Model predictions are adjusted for index case age, sex and ethnicity, contact index of multiple deprivation and incidence as in Figure 2. Abbreviations: Ct, cycle threshold; PCR, polymerase chain reaction; SGTF, S gene target failure.

Adjusted multivariable analysis showed strong evidence of effect modification (interactions) and non-linear relationships, such that associations are best described graphically (Figures 2 and 3, Supplementary Figures 7–11). Index case Ct value was an important determinant of PCR-positive results in contacts, with an approximately linear decline as Ct value increased, which was independent of the nature of the contact event (Figure 2). For example, amongst household contacts, with other variables set to median values/baseline categories, rates of PCR-positive tests were 11.7% (95% CI 11.5–12.0%) for index case Ct = 15 and 4.5% (95% CI 4.4–4.6%) for Ct = 30. Contacts were most likely to test PCR-positive after household contact (percentage of PCR-positive tests, at median Ct value = 20.1, 8.7% [95%CI 8.6–8.9%]), followed by visitors to households (7.1% [95% CI 6.8–7.3%]), contacts at events/activities (5.2% [95% CI 4.9–5.4%]), then work/education (4.6% [95% CI 4.4–4.8%]), with outdoor contacts least likely to test positive (2.9% [95% CI 2.3–3.8%]).

SGTF was associated with increases in contacts testing PCR-positive, by 1.55-fold (95% CI 1.49–1.60) at index Ct = 15, 1.55 (95% CI 1.49–1.61) at Ct = 20 and 1.44 (95% CI 1.38–1.51) at Ct = 30. At Ct values near the upper limit of the assay, the relative increase in PCR-positive results fell to near 1 (Figure 3).

**Figure 4.**
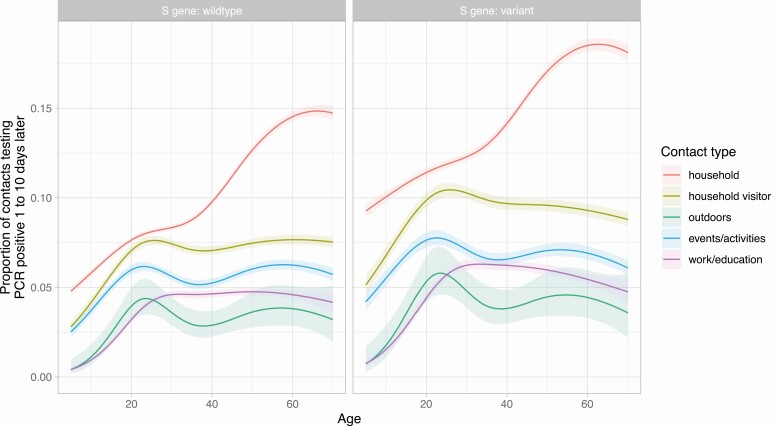
Relationship between index case age and the proportion of their contacts with a PCR positive result, by contact type and S gene target failure. Model predictions are plotted after adjustment for Ct value (set to the median Ct value, 20.1), and other variables as in Figure 2. Abbreviations: Ct, cycle threshold; PCR, polymerase chain reaction.

Contacts of children were the least likely to test positive, particularly following contact outdoors or at work or in education (Figure 4). Most contact types had similar rates of PCR-positive results across adult ages, except for household contact where risk increased as age increased above 35 years and contact at work/education, events/activities and outdoors where risk of a PCR-positive result was highest in adults in their 20s.

Associations between PCR-positive results in contacts and sex varied with age (Supplementary Figure 8). Broadly, increasing incidence increased PCR-positive contacts, likely reflecting increased acquisition from third parties. There were fewer PCR-positive contacts in areas of greater social deprivation (Supplementary Figure 11) and among Black, Asian, and minority ethnic groups (Supplementary Figures 9 and 10).

A sensitivity analysis supported the 1–10 day follow-up window for PCR results in contacts as the association between index case Ct and positive contacts attenuated beyond 10 days, consistent with more acquisition from third parties. There were relatively few contacts testing positive 11–14 days later (Supplementary Figure 12). Case Ct values explained only a small proportion of the variability in contact Ct values (unadjusted linear regression coefficient 0.14 [95% CI .13–.14, *P* < .001], *R*^2^ = 0.02).

### Predictors of PCR-Positive Results in Contacts Attending PCR Testing

In a sensitivity analysis restricted to contacts who had a PCR test (Supplementary Table 2, Supplementary Figures 13–19), similar relationships were seen between PCR-positive results and index case Ct values, contact type, and SGTF (Supplementary Figure 19). Although rates of PCR-positive results remained highest in older adult household contacts, there was attenuation of the lower rates seen in children, consistent with main analysis findings of less transmission from children arising from less testing being required or undertaken in contacts of children (Supplementary Figure 18). In contrast to the main analysis, contacts of all non-white ethnic groups (Supplementary Table 2) and those living in more deprived areas (Supplementary Figure 17) were more likely to be PCR-positive, potentially due to differences in access to and use of testing by different ethnic and socioeconomic groups.

### Proportion of Cases With PCR-Positive Contacts Detected by LFDs

Overall, 85.4% (197 677/231 497) of case-contact pairs with PCR-positive contacts, that is, plausible onward transmission, had estimated case viral loads of ≥10 000 RNA copies/mL (ie, Ct ≤ 24.4) versus 75.2% of all cases (800 020/1 064 004). Index cases with SGTF had lower Ct values, except for results near the detection threshold (Supplementary Figure 20).

**Figure 5.**
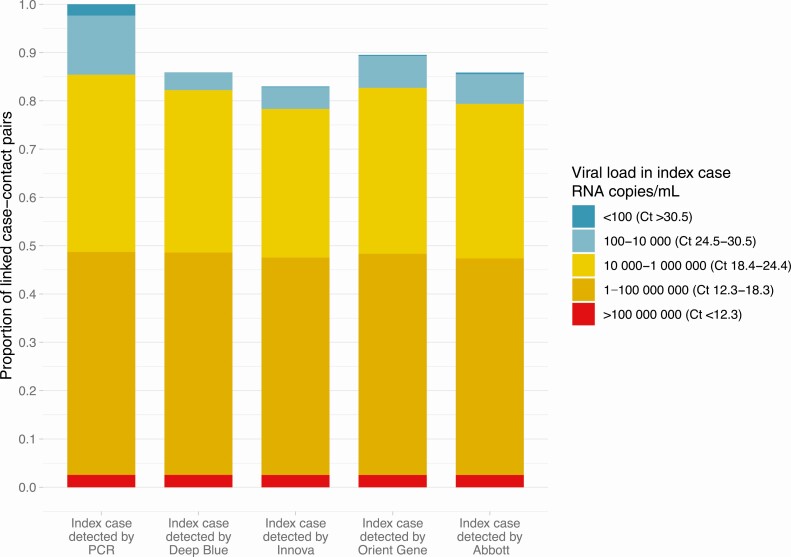
Simulated proportion of cases with a PCR-positive contact detected using 4 LFDs. Proportion of cases detected by estimated PCR viral load (PCR cycle threshold, Ct value) is shown in the PCR column. Number of cases with a PCR-positive contact who would be detected using each LFD is shown for 4 LFDs. Abbreviations: Ct, cycle threshold; LFD, lateral flow devices; PCR, polymerase chain reaction.

As antigen LFD sensitivity varies by viral load, we used the distribution of Ct values in case-contact pairs with a PCR-positive contact to simulate the proportion of such cases who would have been detected using antigen LFDs (Figure 5). The Deep Blue LFD would have detected 85.9% (95% CI 85.8–86.0%) of cases who plausibly subsequently transmitted to a contact, the Innova LFD 83.0% (95% CI 82.8–83.1%), the Orient Gene LFD 89.5% (89.4–89.6%) and the Abbott LFD 85.8% (95% CI 85.7–86.0%). Performance was very similar before and after the B.1.1.7 expansion (Supplementary Table 3). The performance characteristics required to detect varying proportions of transmission sources by a novel LFD are illustrated in Supplementary Figure 21.

## DISCUSSION

We have performed a large-scale analysis of combined SARS-CoV-2 contact tracing and testing data from England involving >2 million contacts of PCR-confirmed cases. We show SARS-CoV-2 infectivity is associated with index case viral load, including after adjustment for demographic factors and type of contact event. SGTF, a proxy for the B.1.1.7 variant, increased transmission by ~50% at most viral loads. Onward transmission from children was relatively uncommon compared to adults, although this may partly be due to less testing in their contacts. We confirm earlier findings that household contact is associated with greater rates of transmission compared to workplace, educational or recreational contact outside of homes [19, 20].

Except SGTF, it is noteworthy that we found no evidence of significant interactions between Ct values and any other variables in the analysis, that is, the effect of viral load on infectivity is generalisable across populations and settings. These results are consistent and add to a recent smaller cohort study [21].

Consistent with other reports [3] we found that SGTF increased the proportion of contacts testing PCR-positive, by around 55% at high viral loads (Ct values of 10–20), rising with moderate viral loads to a maximum of 75% (Ct = 25) before declining again to below 10% at low viral loads (Ct = 34). SGTF also affected how the likelihood of transmission varied with age, contact event type and ethnicity. The higher relative infectiousness at moderate viral loads may represent increased infectiousness of individual virions at viral loads where stochasticity is more important compared to higher viral loads. The attenuation of the relative infectiousness at high Ct values partly arises from greater numbers of wildtype strains exhibiting SGTF due to stochastic failure to detect a single gene at low viral loads. As lower viral loads are less infectious, it may also reflect more PCR-positive contacts acquiring infection from third parties, such that the characteristics of the index case matter less. This is supported by the proportion of contacts testing PCR-positive not tending to zero at very low viral loads.

Overall, 85.4% PCR-positive contacts had an index case with an estimated viral load of ≥10 000 RNA copies/ml (Ct ≤ 24.4). Hence, 85.4% of infections in contacts are potentially attributable to the 75.2% of cases overall with a viral load of ≥10 000 RNA copies/mL. Although differential interventions to prevent onward transmission could be focused on those with high viral loads, our findings suggest that most infected individuals still have some risk of transmitting onwards based on Ct values.

However, we show that several LFDs could have detected most cases that led to onward transmission. These tests offer potential advantages, returning results in 15–30 minutes, not requiring laboratory infrastructure and costing significantly less than PCR. However post-analytic infrastructure is still needed to collect results. Using the estimated sensitivity by viral load of 4 LFDs, we estimate they would detect 83.0%–89.5% of cases leading to onward transmission. Although such performance is not sufficient to replace PCR for testing of all symptomatic individuals, use of LFDs in addition to existing testing, particularly of those who otherwise would not be tested at all (including those without symptoms), would allow many of the most infectious individuals to be identified earlier, potentially preventing onward transmissions and helping to drive reproduction numbers below 1, despite imperfect performance against PCR. The specificity of each LFD is another important consideration, particularly as incidence falls; the false positive rate for the Innova LFD has been previously reported as 0.32% (95% CI .20–.48%),^18^ and large-scale evaluations of the other LFDs are ongoing. In settings where the positive predictive value of an LFD is insufficiently high, confirmatory PCR testing may be required.

Our study has important limitations. First, ascertaining infection in contacts depends on the contact being reported by the case and the contact being tested. In the United Kingdom, PCR testing was only recommended if symptomatic, and therefore we do not ascertain most asymptomatic infections. Although Ct values are generally slightly lower in those without symptoms [22], they may nevertheless contribute substantially to transmission [23]. Additionally, access to testing depends on social and demographic factors, for example, the relationships between PCR-positive results in contacts and ethnicity varied if we conditioned on contact attendance for testing (Table 2 vs. Supplementary Table 2). Furthermore, contacts who tested positive were tested a median (IQR) 3 (2–5) days after their index case, compared to 2 (1–4) days in contacts who tested negative. It is therefore possible that some negative tests in contacts tested earlier may have been positive if subsequently repeated.

Second, our classification of contact events is relatively simple; for example, we do not have any direct measures of human behaviour, such as proximity or duration of contact. We do not account for the dynamic nature of viral loads over time [24], relying on a single measurement at varying times post infection. Despite this, the time from symptom onset to testing in cases was relatively consistent, median (IQR) 2 (1–3) days, such that measured Ct values plausibly represent similar stages of infection. We use only a single assay to determine Ct values, and it should also be noted that Ct values vary by extraction and PCR platforms and so our findings may equate with different Ct values in other diagnostic workflows. However, to facilitate comparisons with other platforms we use an external calibrant to estimate viral load from Ct values, although digital PCR may allow this to be done with more precision.

It was not possible to account for unobserved third-party transmission, although we designed our study population to minimise this risk. This likely means that some contact events identified as possible transmission events may actually not be the source of the infection in the contact. It is likely that proportionally this effect is greatest at lower viral loads (higher Ct values), as the likelihood of transmission rises with viral load.

In summary, we provide strong evidence that SGTF increases SARS-CoV-2 transmission and that SARS-CoV-2 infectivity increases with increasing viral load. We show that the relative strength of the effect of viral load is consistent across ages, ethnicities, and different types of contact events. Despite this association, most individuals have Ct values compatible with onward transmission [25]. Nevertheless, LFDs can detect most individuals who are potential transmission sources. This supports wider use of LFDs as rapid and regular screens to detect infectiousness in populations at high risk of acquisition, including recent contacts of cases. Further prospective studies will be required to demonstrate whether targeted isolation and/or contact tracing, together with wider use of LFDs in combination with vaccination are effective in preventing ongoing SARS-CoV-2 transmission.

## Supplementary Data

Supplementary materials are available at *Clinical Infectious Diseases* online. Consisting of data provided by the authors to benefit the reader, the posted materials are not copyedited and are the sole responsibility of the authors, so questions or comments should be addressed to the corresponding author.

ciab421_suppl_Supplementary_Materials

## Notes

***Disclaimer.*** The funder of the study provided access to the data and facilitated data linkage. The funder had no role in study design, data analysis, data interpretation, or writing of the report. The corresponding author had full access to all the data in the study and had final responsibility for the decision to submit for publication

***Financial support.*** This work was supported by the UK Government’s Department of Health and Social Care. This work was supported by the National Institute for Health Research Health Protection Research Unit (NIHR HPRU) in Healthcare Associated Infections and Antimicrobial Resistance at Oxford University in partnership with Public Health England (PHE) (NIHR200915), and the NIHR Biomedical Research Centre, Oxford. The views expressed in this publication are those of the authors and not necessarily those of the NHS, the National Institute for Health Research, the Department of Health or Public Health England. L. Y. W. L. is supported by the NIHR Oxford BRC. A. S. W. is an NIHR Senior Investigator. D. W. E. is a Robertson Foundation Fellow and an NIHR Oxford BRC Senior Fellow.

***Potential conflicts of interest.*** L. Y. W. L. reports speaker fees from the Merck group and Servier, outside the submitted work. D. W. E. reports lecture fees from Gilead, outside the submitted work. M. P. and S. R. report employment from Accenture. S. R. reports that DHSC were supplied professional services by Accenture. All other authors report no potential conflicts.
